# Influence of bone density on morphologic cement penetration in minimally invasive tibial unicompartmental knee arthroplasty: an in vitro cadaver study

**DOI:** 10.1186/s13018-019-1376-6

**Published:** 2019-10-22

**Authors:** Christian B. Scheele, Matthias F. Pietschmann, Christian Schröder, Igor Lazic, Thomas M. Grupp, Peter E. Müller

**Affiliations:** 1 Department of Orthopaedics, Physical Medicine and Rehabilitation, University Hospital, LMU Munich, Munich, Germany; 2 Department of Orthopedics and Sports Orthopedics, Klinikum rechts der Isar, Technical University Munich, Ismaninger Str. 22, 81675 Munich, Germany; 3 Aesculap AG Research & Development, Am Aesculap-Platz, 78532 Tuttlingen, Germany

**Keywords:** Unicompartmental knee artrhroplasty, UKA, Bone density, BMD, Cementation, Minimally invasive

## Abstract

**Background:**

Unicompartmental knee arthroplasty is an established treatment option for anteromedial osteoarthritis. However, large registry studies report higher rates of aseptic loosening compared to total knee arthroplasty. The objective of this study was to assess the impact of bone density on morphological cement penetration. Moreover, an alternative regional bone density measuring technique was validated against the established bone mineral density assessment.

**Methods:**

Components were implanted on the medial side of 18 fresh-frozen cadaver knees using a minimally invasive approach. Bone density has been quantified prior to implantation using Hounsfield units and bone mineral density. Morphological cement penetration has been assessed in different areas and was correlated with local bone density.

**Findings:**

A highly significant correlation between Hounsfield units and trabecular bone mineral density was detected (*r* = 0.93; *P* < 0.0001), and local bone density was significantly increased in the anterior and posterior area (*P =* 0.0003). The mean cement penetration depth was 1.5 (SD 0.5 mm), and cement intrusion into trabecular bone was interrupted in 31.8% (SD 23.7%) of the bone-cement interface. Bone density was correlated significantly negative with penetration depth (*r* = − 0.31; *P =* 0.023) and positive with interruptions of horizontal interdigitating (*r* = + 0.33; *P =* 0.014). Cement penetration around the anchoring peg was not significantly correlated with bone density.

**Interpretation:**

Areas with high bone density were characterized by significantly lower penetration depths and significantly higher areas without cement penetration. Anchoring pegs facilitate cement intrusion mechanically. Regional quantification of bone density using Hounsfield units is a simple but valuable extension to the established determination of bone mineral density.

## Background

In view of excellent functional results, good long-term survivorship, and advantages in terms of cost efficiency, unicompartmental knee arthroplasty (UKA) can make a significant contribution to meet the rapidly growing demand for knee arthroplasty [1–5]. Since the aseptic loosening of the tibial component, misinterpretation of radiolucent lines and cementation errors remain major reasons for revision in UKA [6–9]; improvements of the interface between prosthesis, cement, and trabecular bone, as well as the optimization of minimally invasive surgical techniques, are of outstanding importance [10, 11].

While former studies focused on levers to improve cemented fixation that can be controlled by the surgeon, such as bone bed preparation, cementing technique, or surgical access [11–17], little is known about the impact of bone density, which can hardly be altered, on cemented tibial fixation in UKA.

The goal of this study was to quantify the impact of regional bone density on different morphological aspects of cement penetration in minimally invasive UKA. In addition, the reliable applicability of Hounsfield unit (HU) measurement, which is a simple but precise tool for local quantification of bone density, has been validated against the established method of bone mineral density (BMD).

## Methods

**Fig. 1:**
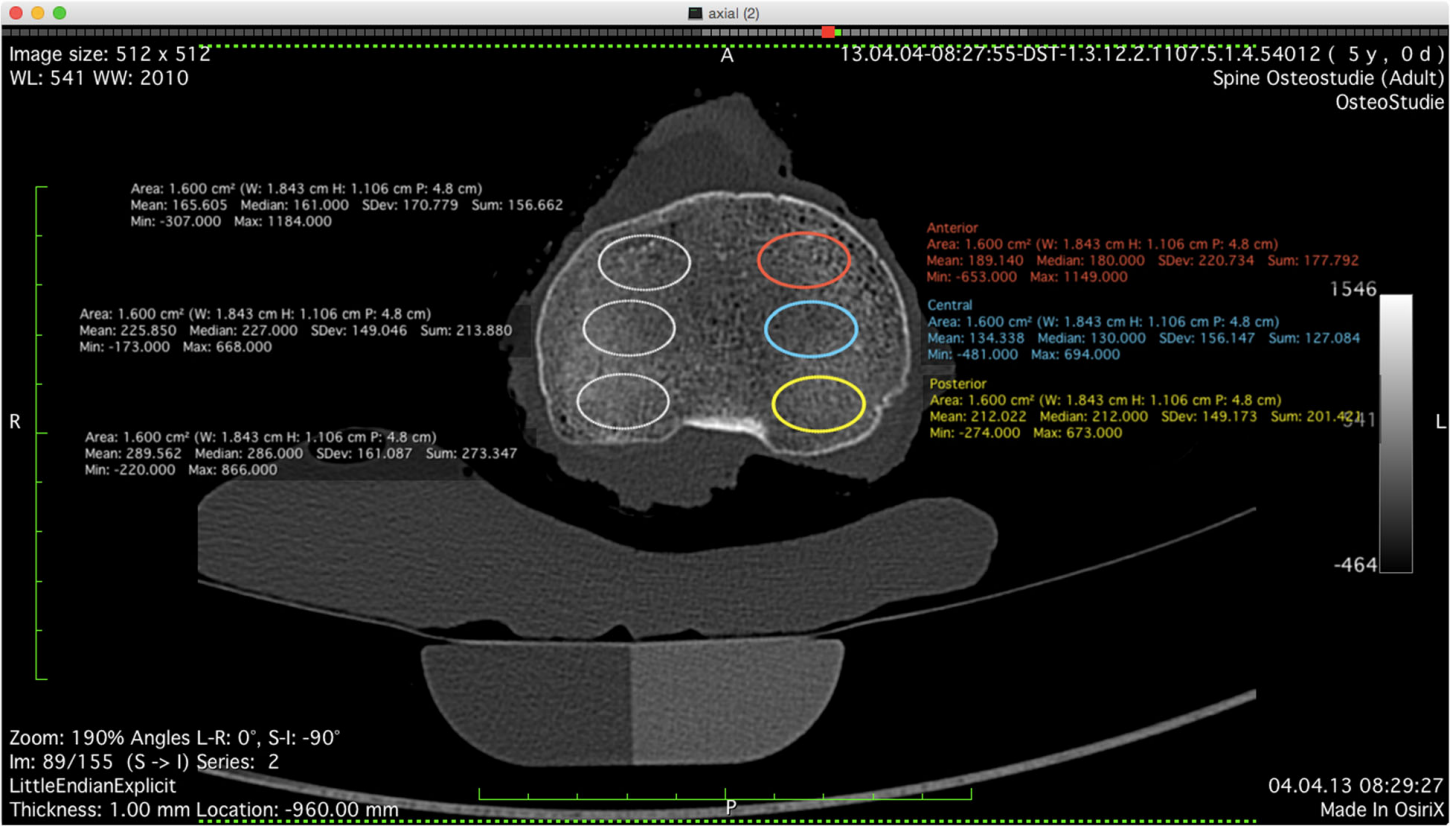
The mean Hounsefield units of six ROIs of 1.6 cm^2^ each, which were strictly located within the trabecular bone, were measured on each CT slice for correlation with mean BMD. The measurement of local bone density in the anterior (red), central (blue), and posterior (yellow) area of the medial tibial plateau-based mean Hounsfield units was used for correlation analysis between bone density and cement penetration pattern

Eighteen fresh-frozen human cadaver knees (age 72.2, SD 14.9 years, 4 females, 14 males) were used for this in vitro study. CT scans (Sensation 64 Somatom, Siemens AG Munich, Germany) of all tibiae were acquired prior to the implantation in order to exclude specimens with osseous abnormalities and to quantify BMD. Cortical and trabecular BMD [milligrams (Ca^2+^HA)/milliliter] were determined using Syngo Osteo CT software (Siemens AG Munich, Germany) on the proximal tibia in seven layers each in 3 mm, using a relative calibration to water (0 HU) and calcium (200 HU). Correspondingly, local bone density was determined by measuring the mean HU of six regions of interest (ROI) per slice, 1.6 cm^2^ each, in the anterior, central, and posterior area of the medial and lateral tibial plateau (Fig. 1). To capture the relevant area of implantation, both evaluations started in the most cranial slice without visible cortical bone or subchondral sclerosis and assessed 2 cm distally.

UKAs were implanted on the medial side of each knee via a minimally invasive approach without eversion of the patella. Tibial resection was performed with an anatomical posterior slope and the removal of the cartilage in all areas of the tibial plateau was ensured intraoperatively. Bone preparation was performed using pulsatile jet lavage (Pulsavac Plus, Zimmer Biomet, USA) and a high viscosity bone cement (Palacos® R 20 g powder/10 ml monomer, Heraeus Medical Wehrheim, Germany) was mixed manually for cemented fixation. The implantation of the components was performed according to the manufacturer’s instructions (Univation® X, Aesculap Tuttlingen, Germany) and all operations were performed by one experienced orthopedic surgeon. The knees were then positioned in 45° flexion and a spacer was inserted to ensure adequate pressurization during polymerization of the cement.

Afterwards, the surrounding soft tissue was removed and the medial tibial plateaus with the cemented tibial trays were dissected (sagittal plane, at the eminentia intercondylaris; transversal plane, 20 mm below the tibial plateau). The 18 specimens were then imbedded in Technovit 4004 (Technovit 4004, Kulzer GmbH, Hanau, Germany) and cut into 10 frontal slices of identical thickness. Scans with a resolution of 100 pixel/mm made the implant–cement–bone interface accessible for morphologic evaluation using Adobe (Photoshop CS6, Adobe, San Jose, USA). Morphologic indicators of cementation were evaluated on both sides of nine cuts through the specimen. The anterior three serial cuts through the implant–cement–bone interface represented the anterior area, the central three cuts represented the central area and the posterior three cuts represented the posterior area of the medial tibial plateau.

**Fig. 2:**
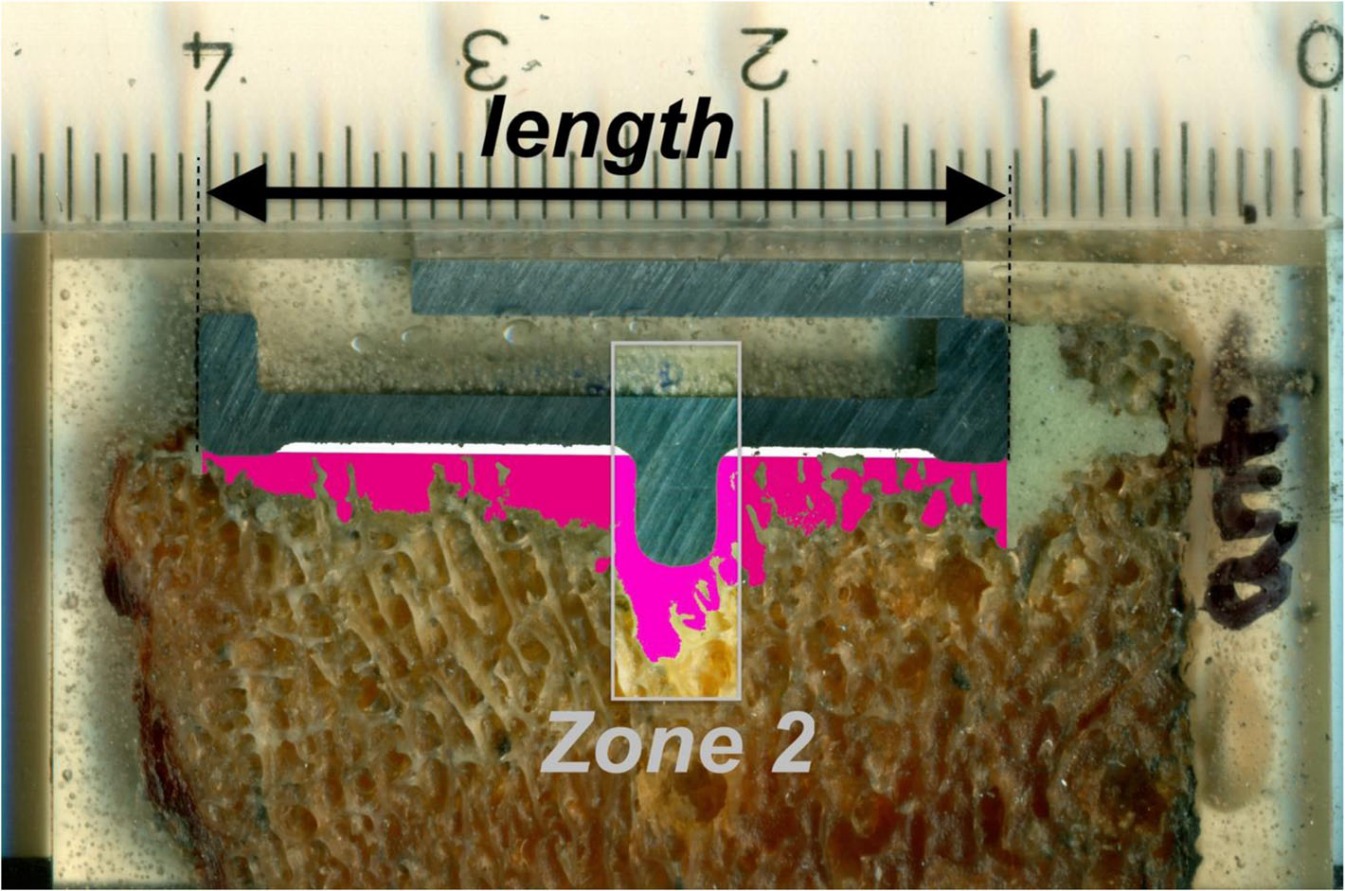
Image of the prosthesis-cement-bone interface: white, cement mantle (above resection line); purple, cement penetration (below resection line). The area adjacent to the anchoring peg was defined additionally as “zone 2”

**Fig. 3:**
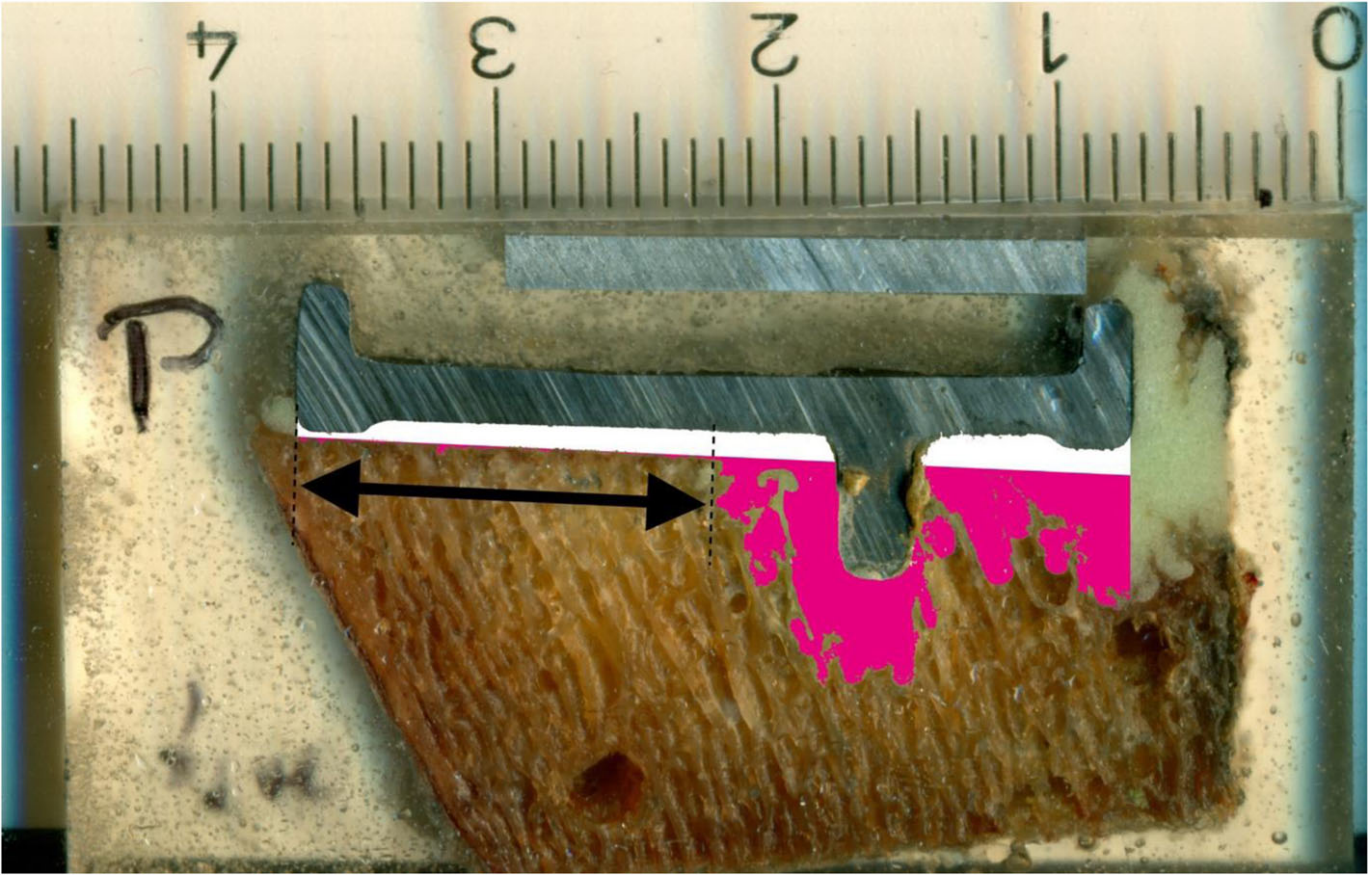
Area without visible cement penetration of bone cement into the trabecular bone in zone 1 (arrow)

The average total cement thickness was calculated by dividing the total area of bone cement by the length of the prosthesis (white and purple area, black arrow in Fig. 2). Cement mantle thickness was calculated by dividing the cement area above the resection line (white in Fig. 2) by the length of the cement-prosthesis interface (defined as contact between the white area and tibial component). Cement penetration depth was calculated by dividing the cement area below the resection line (purple in Fig. 2) by the length of the prosthesis. Furthermore, the length proportion of the horizontal underside of the tibial component (zone 1) without visible cement intrusion into the trabecular bone was determined (Fig. 3). Finally, the proportion of interfaces with visible cement interdigitation in the area adjacent to the anchoring peg (zone 2) of the prosthesis was documented.

For correlation with morphological cement penetration, bone density is quantified using HU, as this allows selective density measurements in the individually investigated subareas of the tibial head.

Results in the text are presented as an arithmetic mean, standard deviation, and minimum and maximum of the evaluated values. To calculate Pearson’s correlation coefficient between BMD and HU, all six ROIs per CT slice were taken into account. Differences in bone density in the different ROIs were analyzed using repeated measures ANOVA and Bonferroni’s multiple comparison test. The effect of bone density on cement penetration was examined by calculating regression lines and Pearson’s correlation coefficients based on all 54 ROIs. All tests were two-sided and a *P* value of 0.05 was considered significant. Statistical analysis was performed with GraphPad Prism 5 (GraphPad Software, Inc., La Jolla, USA).

## Results

**Fig. 4:**
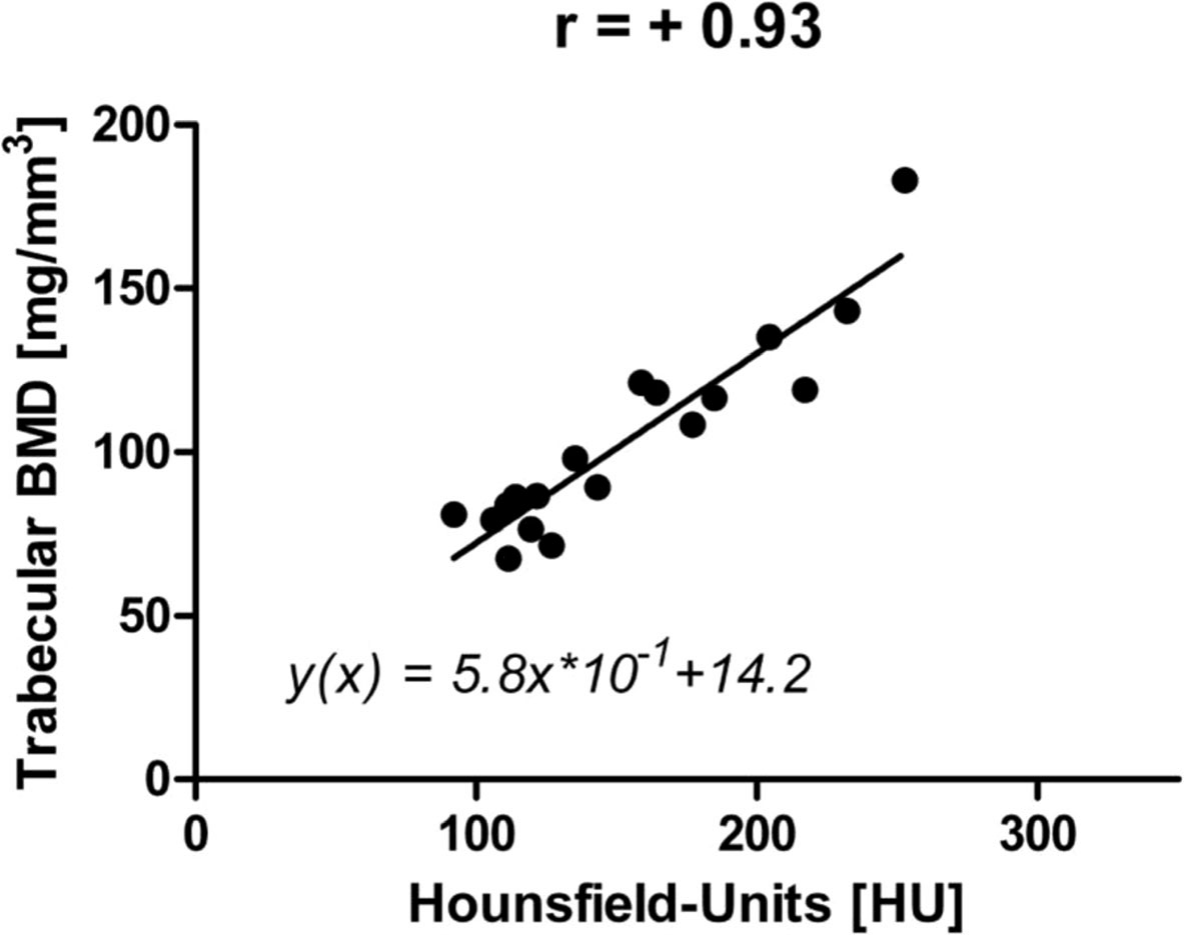
Correlation between BMD and HU

The mean cortical BMD was 327.7 (SD 99.4 mg/ml) [185.8–535.6], mean trabecular BMD was 103.6 (SD 29.5 mg/ml) [67.4–183.0], and mean HU was 154.0 (SD 47.8) [92.0–252.8]. Between the trabecular BMD and HU, a highly significant correlation was detected with a correlation coefficient of *r* = 0.93 (*P* < 0.0001; Fig. 4). The correlation between the cortical BMD and HU was *r* = 0.65 (*P =* 0.0037). The correlation between age and HU was not significant (*r* = − 0.36; *P =* 0.1414).

**Fig. 5:**
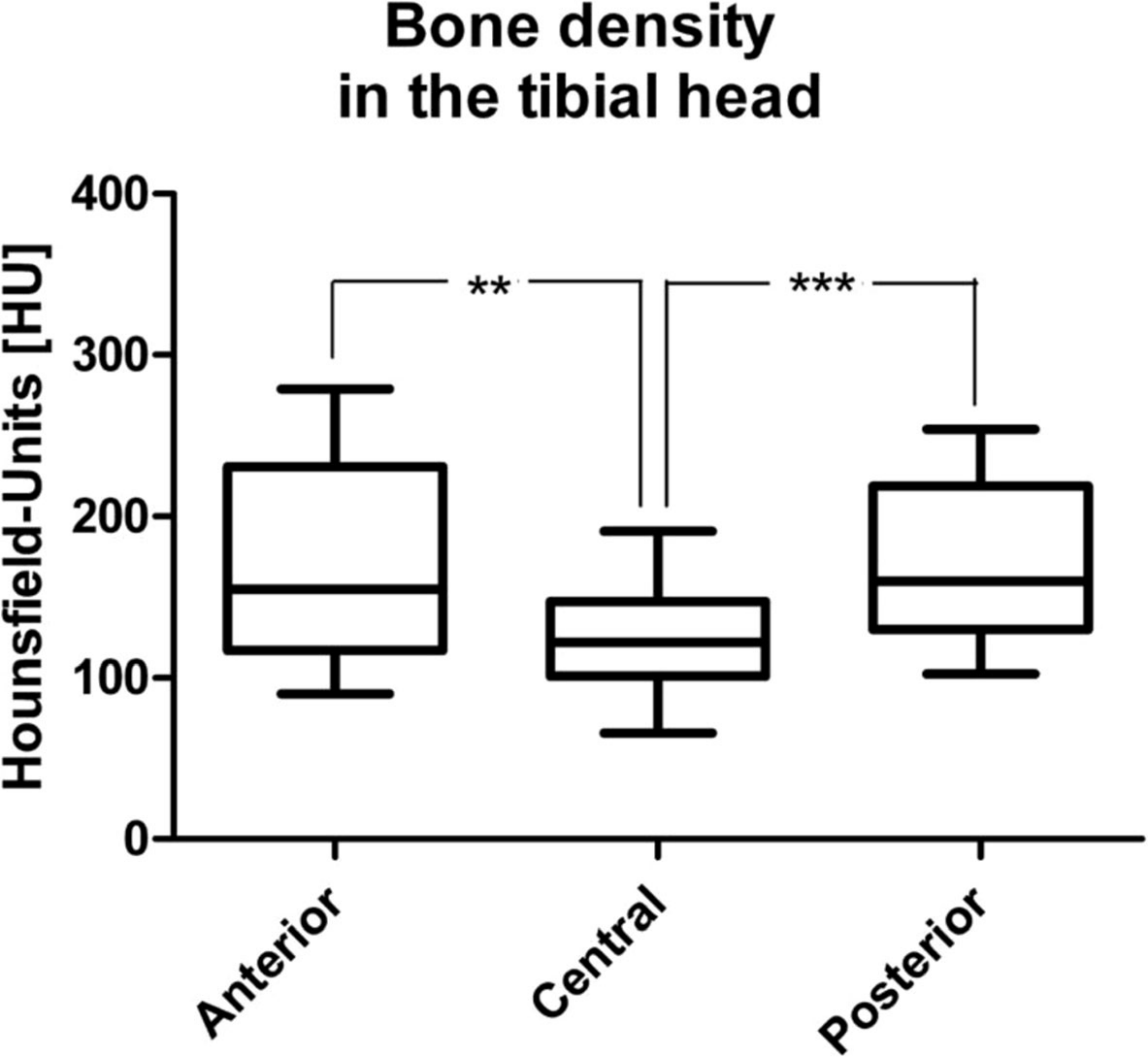
Anterior-posterior development of HU. Whiskers, 10–90 percentile

The mean HU was 168.0 (SD 65.6) [83.1–294.1] in the anterior, 124.1 (SD 41.8) [55.2–215.7] in the central and 175.0 (SD 56.2) [94.3–295.5] in the posterior area of the medial tibial plateau, demonstrating significant differences (*P =* 0.0003). Differences between the anterior and central and between the central and posterior area were significant (*P* < 0.05; Fig. 5).

**Fig. 6:**
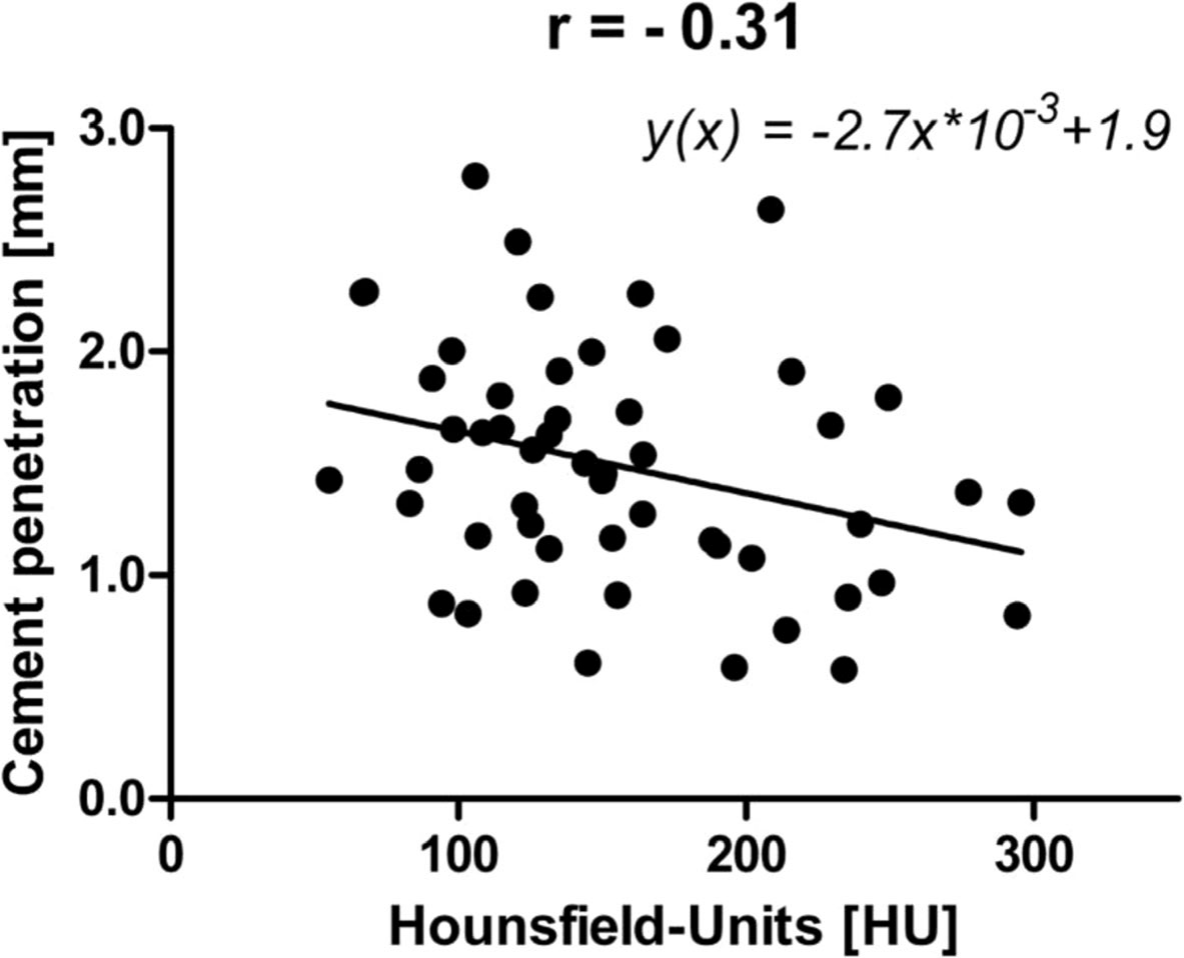
Correlation between bone density and penetration depth in area 1

Based on all 54 ROIs, the mean total cement thickness was 2.1 (SD 0.6) [0.9–3.8], mean cement mantle thickness was 0.8 (SD 0.6) [0.0–2.8], and mean cement penetration depth was 1.5 (SD 0.5) [0.6–2.7]. Only the penetration depth was significantly negative correlated with HU (*r* = − 0.31; *P =* 0.023; Fig. 6). On the contrary, there was no significant correlation between the total cement thickness (*r* = − 0.24; *P =* 0.081) or cement mantle thickness (*r* = 0.05; *P =* 0.70) and bone density (HU).

**Fig. 7:**
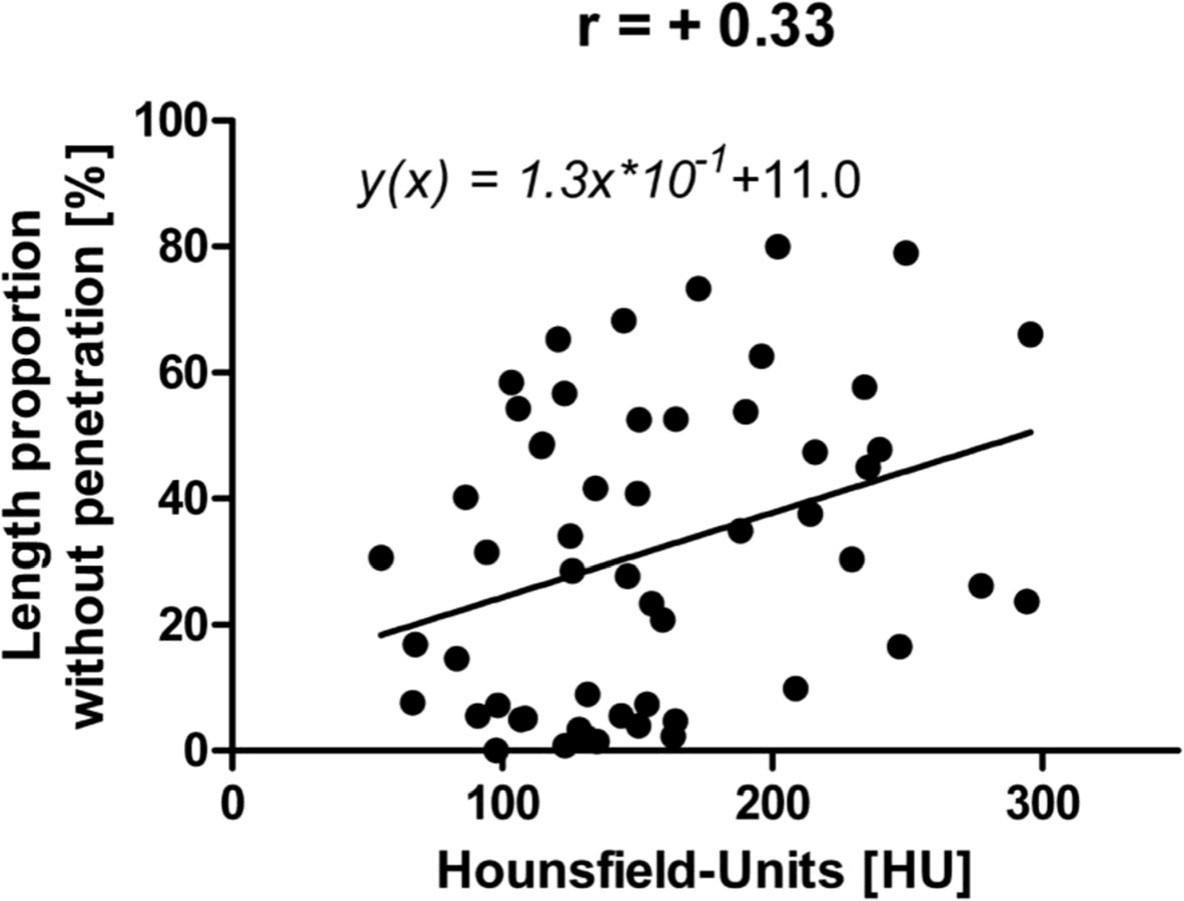
Correlation of bone density with the interruptions of penetration in area 1

**Fig. 8:**
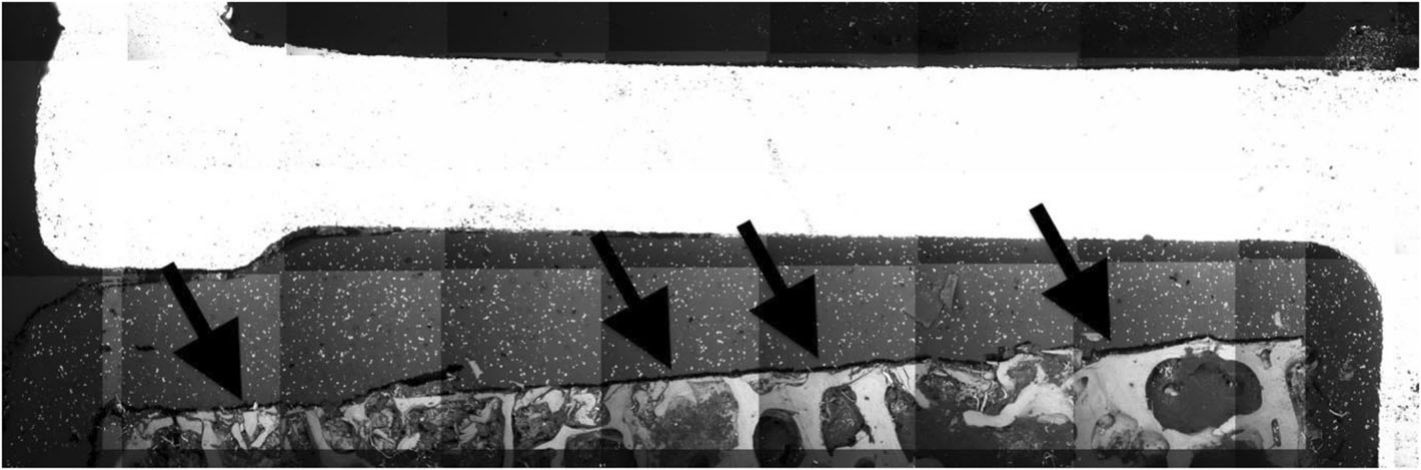
SEM image, an extended area without cement penetration in area 1. Arrows, examples of how high bone density prevents bone cement from penetrating into the interspaces of the trabecular bone and thus prevents a mechanical interlocking

The average interface proportion without cement penetration in zone 1 was 31.8% SD 23.7% [0.0–80.0%]. The correlation between HU and the proportion without cement penetration in zone 1 was *r* = + 0.33 (*P =* 0.014; Fig. 7). Figure 8 shows an SEM image of an implant–cement–bone interface, that provides a morphological example of an extended area without cement intrusion in zone 1 (specimen no. 9, third cut post; approx. 164 HE).

The manifestation of visible cement penetration in the area directly adjacent to the anchoring pegs (zone 2) does not significantly correlate with bone density (*r* = 0.24; *P =* 0.3288). The absence of penetration in zones 1 and 2 does also not correlate significantly with each other (*r* = 0.26; *P =* 0.3044).

## Discussion

In knee arthroplasty, achieving primary stability is crucial to prevent micromotions, which can lead to a vicious circle of membrane formation, micro fractures, radiolucent lines (RLL), and aseptic loosening [18–21]. Both RLLs and aseptic loosening occur earlier on the tibial side than on the femoral side [22, 23], which emphasizes the importance of solid cementation in tibial UKA.

Our results show relevant areas without cement penetration at the bone-cement interface whose occurrence is positively correlated with bone density. In addition, the average cement penetration depth appeared reduced by high bone density. Interestingly, this relationship between bone density and penetration was not found in the surrounding area of the anchoring pegs.

As bone cement has no adhesive properties, fixation is rather achieved by its intrusion into the microstructure of the trabecular bone and its area-wide mechanical interlocking than by a superficial conformity with irregularities of the bone bed [19, 24–26]. In other words, the primary stability of the cemented fixation, measured as resistance to shear and tensile forces, depends on cement penetration into the adjacent trabecular bone, which is therefore is often used as an indicator of biomechanical stability [13, 24, 26–30]. Nagel et al. demonstrated that a mean tibial penetration depth of at least 1.1 mm is required to maximize the fixation strength [31]. The mean cement penetration depth registered in this study was 1.5 (SD 0.5) mm, which is well above this lower limit of penetration depth and in line with former studies [13, 32].

The extent of cement penetration depth results from the interaction between the cementing technique, cement properties, and bone density. Concerning the cementing technique, it is generally accepted that cleaning of the trabecular resection surfaces is a prerequisite for adequate cement penetration [13, 15, 32–34]. The application of cement to a dry trabecular surface that is free of blood, fat, bone marrow, and debris improves the interlocking of the cement and thus the fixing strength [35–37]. With regard to bone cement, penetration tends to increase with the viscosity of the cement [25, 38–40].

In contrast to the previously mentioned aspects, bone density is a factor that cannot be easily controlled by the surgeon. Penetration is expected to decrease with increasing bone density or decreasing porosity of the bone [26, 28, 41]. However, the specific effect of bone density in minimally invasive implanted UKA with limited intraoperative access, thus restricted the potential of bone preparation and component impaction, remains unclear.

The presented assessment of cement penetration on serial cuts and the quantification of bone density via BMD, which has been adapted for extremities in other studies, can be regarded as well-established methods [15, 19, 42–45]. In addition, bone quality was measured via HU in the anterior, central, and posterior part of the tibial plateau.

The highly significant correlation between HU and trabecular BMD underlines the applicability of HU for quantification of bone density (*r* = 0.93; *P* < 0.0001). The use of HU appears attractive as it enables a precise regional quantification of trabecular bone density. In this analysis, it was fundamental for the identification of the significantly increased average bone density in the anterior and posterior area. In combination with the poorer accessibility for jet lavage and the more difficult pressure application, it uncovers the posterior tibial area, analogous to the posterior femoral condyle, as a potential weak point of minimally invasive UKA implantation [11, 15]. As regional HU measurements focused on trabecular areas, the less positive correlation between HU and cortical BMD seems plausible. The negative correlation between age and bone density did not reach the level of significant, which might be attributable to the rather low case number.

Based on three ROIs per specimen and the analysis of 18 UKAs, the morphologic correlation analysis comprises 54 data points. Our results demonstrate a significantly negative correlation between bone density and penetration depth (*r* = − 0.31). These values correspond to the correlation coefficient reported by Askew et al. (*r* = − 0.24) [28]. To about the same extent, bone density restricts area-wide cement intrusion into the trabecular bone (*r* = + 0.33). Figure 7 shows how the high bone density prevents the cement from penetrating into the interspaces of the bone trabecula and thus prevents a planar interlocking.

The question to what extent the minimally invasive surgical approach, despite the use of jet lavage, impedes the opening of the bone substance, or if available instruments do not allow optimal pressurization of the prosthesis against the bone surface, especially in the posterior area, cannot be answered with the available date. In any case, we recommend the use of drill holes or special devices for microfracturing to prepare anchor wholes during the implantation of UKA in a minimally invasive approach.

Anchoring pegs on the undersurface of the prostheses improve cement penetration largely independent from bone density. Our results demonstrate that in contrast to zone 1 (Fig. 8), there is no significant correlation between bone density and cement penetration around the anchoring peg in zone 2. Thus, a mechanically promoted penetration in zone 2 may be assumed. This observation and the low correlation between penetration around the peg and underneath the horizontal part of the component prove the sense of a differentiated assessment of the two zones and might influence future prosthetic design, e.g., by adding further anchoring pegs.

Concerning cement application and pressurization, Dorr et al. suggested manual cement application after mixing cement for less than 3 min and preparing the bone bed with jet lavage [46]. However, Lutz et al. stated in an in vivo study on TKA that the widespread use of manual cement application, despite jet lavage, is not a reliable method to achieve sufficient cement penetration [14]. Experimental in vitro studies also showed that manual cement application leads to lower penetration depths than the use of a cement gun [24]. Due to the minimally invasive approach and the limited surgical field, manual application of bone cement was used in this study and might add to relevant areas without cement penetration in zone 1. In addition, the choice of a highly viscous Palacos® R bone cement might also have had a negative influence on penetration. Noble and Swarts doubted that penetration depths of more than 3 mm are achievable using Palacos and a pressure of 35 kPa, even after excluding all bone specimen with closed intertrabecular spaces [38]. These results are consistent with the highest penetration of 2.7 mm observed in this study.

## Conclusions

Specimens with high bone density are characterized by a significantly higher interface proportion without visible penetration and by significantly lower penetration depths in zone 1. In contrast, bone density does not reduce penetration around the anchoring peg, probably due to mechanical forced pressurization. Going forward, the reported results foster considerations of additional drill holes to open sclerotic bone or the feasibility of pressurized cement application, additional anchoring pegs, or optimized impaction instruments for minimally invasive procedures. Further research is required to assess the balance of bone density and cement penetration with regard to biomechanical stability.

## Data Availability

The datasets used and/or analyzed during the current study are available from the corresponding author on reasonable request.

## Abbreviations

BMD: Bone mineral density
CT: Computed tomography
Fig.: Figure
HU: Hounsfield units
ROI: Region of interest
SEM: Scanning electron microscope
TKA: Total knee arthroplasty
UKA: Unicompartmental knee arthroplasty
